# Remote delivery of culturally adapted prevent-teach-reinforce for families with Chinese American families of young autistic children

**DOI:** 10.3389/fpsyt.2026.1783825

**Published:** 2026-04-21

**Authors:** Jinlan Zhu, Wendy Machalicek, Qi Wei

**Affiliations:** 1 Department of Special Education and Clinical Sciences, University of Oregon, Eugene, OR, United States; 2 Department of Special Education and Communication Disorders, University of Nebraska Omaha, Omaha, NE, United States

**Keywords:** autism, cultural adaptation, parent-mediated intervention, prevent teach and reinforce, telepractice

## Abstract

**Introduction:**

Chinese American families of autistic children remain underrepresented in the autism intervention literature.

**Methods:**

The current study examined the efficacy and social validity of a culturally adapted and telepractice version of Prevent, Teach, and Reinforce for Families (PTR-F) for Chinese American families of young autistic children in the U.S. Two independent randomized multiple baseline designs across six mother-child dyads were used to examine the effects of the culturally adapted PTR-F intervention program when delivered by mothers on the decreased rate of target child challenging behavior.

**Results:**

A clear functional relation was demonstrated between the intervention and increased behavior support plan (BSP) strategy use for all mothers. Following parent education and coaching, all six mothers demonstrated immediate and sustained increases in BSP implementation fidelity, reaching at least 80% fidelity. A clear functional relation was demonstrated between increased parent strategy use and decreased child challenging behavior for two of the six Dyads and weaker but present participant level effects for Dyads 1,2,3, and 4 due to decreasing baseline trends in challenging behavior and lack of clinically significant decreased challenging behavior for Dyad 4. Social validity findings indicated high parent satisfaction with the intervention goals, procedures, and outcomes, as well as strong acceptability of the telepractice delivery model.

**Discussion:**

These findings have implications for the development and delivery of culturally adapted, family centered telepractice intervention to reduce children’s challenging behavior and expand equitable access to evidence-based autism services for underserved populations.

## 1 Introduction

Parents raising a child with autism spectrum disorder (ASD) experience high levels of parenting stress, financial burden, poorer mental health, parent well-being and quality of family life (1, 2). A critical contributor for each of these negative impacts is the presence of child challenging behavior. Parents of children with ASD who engage in challenging behavior report significantly higher levels of stress compared to those without co-occurring conditions (3). Additionally, studies have found a bidirectional positive relation between parent stress and child challenging behavior with heightened stress contributing to worsened challenging behavior (4, 5) making the development of effective parent mediated interventions to address challenging behavior a strategic investment for improving both child and family outcomes.

One such manualized, evidence-based model for preventing and addressing child challenging behavior and enhancing family quality of life is Prevent-Teach-Reinforce for Families (PTR-F, 6). PTR-F is an extension of the Prevent-Teach-Reinforce (PTR, 7) model used in elementary and middle schools and the Prevent-Teach-Reinforce for Young Children (PTR-YC, 8) model designed for preschool and childcare settings. Each PTR program has foundations in applied behavior analysis (ABA) and positive behavior supports (PBS); however, the PTR-F program provides family members, rather than professional educators or behavior specialists, with the support and instruction needed to implement effective BSPs in their homes and communities.

A growing number of single-case experimental design (SCED) studies (9–12) have examined the effects of face to face and telepractice delivered PTR-F with families of young children with ASD and related diagnoses (i.e., developmental delay, language delays with sensory processing issues) as well as children without developmental delays. Across five SCED studies and 11 children, parents increased their implementation of individualized BSP strategies and their children engaged in lower rate challenging behavior and higher rate adaptive behavior. Parents have also consistently rated PTR-F as having high social validity. Despite successful demonstrations of intervention effect, these studies lacked racial/ethnic and sociodemographic diversity (i.e., only included White middle class families).

In a larger-scale study, Argumedes et al. (13) conducted a non-blinded randomized controlled trial comparing PTR-F to a brief parent training program in a racially and linguistically diverse sample of 24 families of children with ASD (i.e., 39% White, 9% Black, and 52% other ethnic backgrounds). Parents in the PTR-F group implemented the intervention with an average fidelity of 91.5% and their children exhibited greater reductions in challenging behavior and increases in adaptive behavior when compared to the brief parent training program.

In a cross-cultural replication, Loh et al. (14) applied PTR-F with two Taiwanese families of children diagnosed with ASD or developmental delay using SCED withdrawal designs. Stay at home mothers from middle-to-lower-income backgrounds demonstrated high implementation fidelity (≥90%) with marked reductions in child challenging behavior and improved efficiency in completing daily routines. Although recent studies on PTR-F have begun to include racially, culturally, and linguistically diverse families, these demonstrations did not include cultural adaptations to address specific beliefs, practices, or barriers.

Like most evidence-based interventions, PTR-F was originally developed and empirically evaluated within Western, White, middle-class contexts, with limited attention to how culturally shaped parenting beliefs, communication styles, and help-seeking norms may influence caregiver engagement and implementation (15–17). Simply applying the original PTR-F model with diverse families without explicit cultural adaptation may limit its contextual fit and sustainability.

To date, only Choi (18) explored the cultural responsiveness of the PTR-F process with families from Korea, Thailand, and Kazakhstan. The study adapted the PTR-F process by introducing the researcher’s own cultural background as a Korean immigrant to the U.S., explicitly discussing the family’s culture and values, and assessing the contextual fit of the BSP. Choi (18) focused largely on the adaptations to the process of the PTR-F, with limited attention given to content such as language, metaphor, content, concepts, and method. However, when considering the barriers faced by culturally diverse families, relying solely on these three steps might be insufficient for a comprehensive adaptation of an intervention as cultural practices differ across Asians. Despite Choi’s cultural awareness and practice of cultural humility, there still existed a cultural gap between Choi and the non-Korean families.

To address these inequities, including the historical exclusion of racially and ethnically diverse families from interventions such as PTR-F, the lack of systematic content-level cultural adaptations for Asian families, and the cultural mismatches that arise when interventions are implemented across cultures without intentional modification, one approach is to culturally adapt original evidence-based interventions. Cultural adaptation is defined as “the systematic modification of an evidence-based treatment or intervention protocol to consider language, culture, and context in such a way that it is compatible with the client’s cultural patterns, meanings, and values” (19, p.362). The current SCED study adapted the PTR-F model for Chinese American families of young children with ASD in the U.S., using Bernal et al.’s (20) Ecological Validity Model (EVM) as a guiding framework to adapt eight culturally relevant dimensions of the intervention—language, persons, metaphors, content, concepts, goals, methods, and context. These adaptations are essential because cultural context shapes how caregivers define, respond to, and prioritize challenging behaviors, which may in turn affect the selection, implementation, and outcomes of behavior reduction strategies (21). For example, Wei et al. (22) found that among Chinese caregivers of children with ASD, endorsement of *shaming*, a culturally specific child-rearing value involving the use of reprimands and expressions of disappointment to shape behavior, was associated with greater acceptability of punishment-based strategies, whereas endorsement of *training* predicted higher acceptability of positive behavior support approaches. These findings suggest that even when evidence-based behavior reduction strategies are equally effective, caregivers’ cultural values may influence which procedures they find acceptable and are willing to implement consistently. To increase accessibility for a population facing a shortage of English-Mandarin bilingual behavior specialists, the intervention was delivered remotely using telepractice (e.g., 10, 14). The study addressed the following research questions:

RQ1. Is there a functional relation between implementation of the culturally adapted PTR-F parent education and coaching intervention package and increased level of parent BSP strategies implementation during desired family routines?

RQ2. Is there a functional relation between increased level of parent implementation of BSP strategies and decreased level of child mild-moderate challenging behavior during desired family routines?

RQ3 and RQ4. Do parents perceive the goals, procedures, and outcomes of the culturally adapted PTR-F parent training program as acceptable, feasible and effective? Do parents perceive the goals, procedures, and outcomes of the remote telepractice technologies used to deliver the culturally tailored PTR-F program as acceptable, feasible and effective?

## 2 Method

### 2.1 Participants

#### 2.1.1 Recruitment

Caregivers were recruited over three days from flyers posted on Chinese social media applications Xiaohongshu and WeChat. From 56 potential participants who contacted the first author, phone and/or zoom screenings (procedures available from first author) were conducted with the first 21 interested parents residing in the U.S. To confirm presence of child challenging behavior necessitating intervention, potential caregiver participants completed the 99-item Child Behavior Checklist for ages 1 ½-5 (CBCL, 23) to indicate the intensity of their child’s internalizing, externalizing, and total challenging behavior (see **Table 1** for participants’ summary scores). The CBCL has adequate discriminant and convergent validity and is sensitive to changes in challenging behavior (24). The CBCL has been translated into Chinese versions (25), which was used in this study for three of the six parents.

**Table 1:** Child behavior checklist for ages 1.5-5 results for each participant.

Participant	Internalizing		Externalizing		Other problems	Total Problems Score	
	Score	*T-score*	Score	*T-score*		Score	*T-score*
Jie	25	70	23	61	32	80	70
Yiyi	9	56	15	56	12	36	58
Wei	42	91	38	68	39	119	90
Lanlan	16	64	29	67	18	63	67
Ningfeng	28	75	37	68	33	98	79
Meisheng	47	93	50	78	46	143	93

#### 2.1.2 Mother child dyads

From the resultant nine parents meeting inclusion criteria, the first author selected six parent-child dyads meeting inclusion criteria for participation and three parents remained on a waitlist for study duration in case of attrition. The six participating parents were biological, married mothers between the ages of 32 and 49 years of age (M = 38.33 yrs) with Mandarin as their heritage language; however, each family used both Mandarin and English at home. Each mother had lived in the U.S. an average of 11.83 years (Range = 8–19 years). Caregivers reported their child with ASD engaged in mild to moderate challenging behavior during daily family routines and had not received professional guidance to address challenging behavior at home. Except for Lanlan who was a girl, child participants were boys. Except Yiyi, each child scored 60 or higher on the Externalizing Score or Total Problems Score suggesting need for the intervention. Although Yiyi’s CBCL results did not meet clinical threshold of scores of 60 or higher on the Externalizing Score or Total Problems Score), Yiyi’s raw score of 10 on the CBCL Attention Problems subscale was in the borderline clinical range and his mother noted daily disruptive behavior. The interventionist observed Yiyi and his mother confirming challenging behavior appropriate for the intervention. **Table 2** provides additional participant demographics and **Table 3** provides targeted family routines and target challenging behavior. To identify one daily family routine disrupted by challenging behavior, the interventionist interviewed each parent before baseline assessment using the Routines-Based Interview (RBI, 26) which asks parents to rate how each discussed routine is going using a terrible to fantastic scale (1 = terrible; 5 = fantastic). Target challenging behavior was selected based on routine selection.

**Table 2:** Parent-child dyad characteristics including child age, diagnosed age, and family members living with child and mother age, years residing in the U.S., highest education level achieved, current employment and preferred language during the study.

Child	Jie	Yiyi	Wei	Lanlan	Ningfeng	Meisheng
Age	4 years 9 months old	4 years 4 months old	3 years 6 months old	4 years 5 months old	4 years 7 months old	4 years 4 months old
Diagnosed age	3 years old	2 years old	2 years old	2.5 years old	2.5 years old	3 years old
Family members living with child	Dad, Mom, grandparents (Mom’s parents), elder sister, Jie, young sister, Aunt (Mom’s sister)	Grandma (Mom’s mom), Mom, Yiyi (Dad and older brother in China)	Dad, Mom, Grandma (Dad’s mom), Wei, young sister	Dad, Mom, Lanlan, young brother	Dad, Mom, older brother, Ningfeng	Dad, Mom and Meisheng
Parent	Ling	Minyue	Xiaoxiao	Ting	Nana	Yuanyuan
Age	39	37	35	38	49	32
Years in US	19	11	9	16	8	8
Education	High school	Master	Master	Doctorate	Associate degree	High school
Current employment	Unemployed	Risk professional	Unemployed	Data scientist	Unemployed	Unemployed
Preferred language	Mandarin	English or Mandarin	Mandarin	English or Mandarin	Mandarin	Mandarin
SL-ASIA score	53/130	60/130	54/130	54/130	54/130	49/130
Key cultural values	Environmental attribution (COVID-19); strong emphasis on family harmony; value on child obedience	Initial diagnosis denial; environmental causation belief (river pollution); maternal self-blame consistent with collectivist norms	Environmental (COVID-19) and genetic attributions; collectivist family values	Strong collectivist orientation; heightened concern about public “face”;Strong emphasis on child compliance and respect for authority	Autism viewed as potentially curable illness; strong stigma and family reputation concerns; heightened concern about child’s behavior in public and “losing face”; emphasis on following rules and respecting authority	Value on academic achievement and rule-following; concerns about losing face due to child’s behavior in school and public; concern about teacher negative feedback
Family cultural context → Adaptation implications	Grandparents primarily supported typically developing siblings; mother assumed sole responsibility for autistic child within extended family structure; delayed help-seeking and alternative treatment pursuit (cord blood) → Evidence-based psychoeducation	Paternal grandparents questioned mother’s caregiving; sought second diagnosis in China; father and older sibling reside in China; grandparents preferentially support typically developing sibling; mother is the sole caregiver in U.S. → Reinforcing maternal implementation efforts and confidence-building	Paternal grandmother questioned caregiving and primarily supported typically developing sibling; grandparents and husband desired another son, a healthy son to carry on the family name; marital stress present → Maternal self-efficacy and structured mother-led routines	Grandparents primarily supported typically developing sibling; quickly accepted the ASD diagnosis upon receiving it; mother highly educated (doctorate) → Play-based routine selected to enhance interaction, motivation, and parent–child connectedness	Father blamed mother for child’s challenging behavior, exacerbating caregiver stress; family avoided returning to China for 8 years due to stigma → Stigma-sensitive reframing and caregiver support emphasis	Father first identified child’s concerns; grandparents in China with limited influence on daily caregiving; relatively higher paternal involvement → Focus on home–school consistency and refusal behavior during homework

Dyad 1: Jie and Ling. Jie was a 4-year-9-month-old boy who engaged in challenging behavior (i.e., protests, tantrums, leaving area) during homework time in the main living area of the family’s apartment afterschool. His classroom teacher assigned daily homework included counting, coloring, writing numbers and letters, and cutting with scissors. His mother assigned additional homework including writing his name and activities related to teacher-assigned work. He primarily used English at home, relying on single English words, occasional Mandarin phrases, and leading adults by hand to request items and activities.

Dyad 2: Yiyi and Minyue. Yiyi was a 4-year-4-month-old boy who engaged in challenging behavior (i.e., protests, leaving area, crying or whining) during playtime which occurred in the common area of the house after dinner with Yiyi’s grandmother sometimes present. Playtime was the daily routine in which Minyue was most actively engaged and could intentionally guide Yiyi’s behavior toward targeted skills. Minyue planned fine motor, sensory play or pretend play activities (e.g., threading beads, playdough, animal farm) in this routine. Yiyi mainly spoke Mandarin at home with insufficient intelligibility and did not spontaneously use language to request or protest items and activities, which contributed to misunderstandings and tantrums. For instance, if he put on his shoes and ran to open the door to outside, his mother would say, “我要出去玩” (“I want to go out to play”) and would only open the door contingent on his request. Although playtime is often associated with child-preferred, unstructured contexts, Minyue planned structured, adult-directed activities requiring Yiyi to sit at a table and comply with activity rules.

Dyad 3: Wei and Xiaoxiao. Wei was a 3-year-6-month-old boy who engaged in challenging behavior (i.e., protests, tantrum, leaving area) during occupational therapist assigned exercise (i.e., at least 10 min. daily running on treadmill) which was conducted in a separate space of the family’s apartment with Wei and his younger sister following his afterschool nap. There was a bike, balance bike, scooter, circle for jumping, balance beam, treadmill, basketball, football, and bouncy ball in the exercise area. They were occasionally joined by their father and/or grandmother. Wei used both English and Chinese fluently at home but mainly requested in complete sentences in English.

Dyad 4: Lanlan and Ting. Lanlan was a 4-year-5-month-old girl who engaged in challenging behavior (i.e., inattention, disengagement, leaving area) during evening playtime which occurred in the family’s office which included a working table, computer, sofa, bouncing ball, and toys. Playtime involved educational activities such as letter, number, and shape puzzles, matching games, and tablet-based activities. Mandarin was mostly spoken at home, but Lanlan mostly played alone and rarely initiated communication. She answered yes or no questions and primarily made requests using multiword Mandarin phrases such as “吃西瓜” (“eat watermelon”), “喝粥” (“drink porridge”), but did not answer more complex or open-ended questions.

Dyad 5: Ningfeng and Nana. Ningfeng was a 4-year-7-month-old boy who engaged in challenging behavior (i.e., leaving area, tantrum) during table work, which took place in the main living area of their apartment afterschool. Nana planned educational activities such as reading, or writing his name, numbers, and letters. When not engaged in screen time, Ningfeng often engaged in running and jumping, making focus on table tasks or games difficult. The family primarily communicated in Mandarin at home, but Ningfeng’s communication was limited and primarily included leading others by hand.

Dyad 6: Meisheng and Yuanyuan. Meisheng was a 4-year-4-month-old boy who engaged in challenging behavior (i.e., protests, tantrums) during homework time which occurred in a separate room of their apartment afterschool. Yuanyuan planned educational activities such as reading, writing his name, numbers and letters, and playing games. The family communicated in both English and Mandarin at home. While Meisheng understood Mandarin, he more often used English to request when prompted to use two to three-word phrases (e.g., “read a book,” or “do exercise,”).

This research received approval from the University of Oregon Institutional Review Board (IRB) and each mother provided informed consent and child assent was waived by the IRB due to participants’ age and communication limitations.

### 2.2 Settings and materials

The researcher initiated all sessions via telepractice from a private working space (i.e., a lockable door to prevent unexpected entry). Sessions were conducted via HIPAA-compliant Zoom provided by university with parents and the interventionist using personal computers or tablets paired with internal or external webcams, and Bluetooth headphones to engage in synchronous audio and visual transmission. Before each session, the interventionist assisted parents in setting up their devices to allow unobstructed views of parent–child interactions during selected family routines, enabling real-time observation, coaching, and feedback. Security features included waiting rooms, restricted access, and renaming participants using pseudonyms. Sessions were recorded and transferred to a HIPAA-compliant OneDrive folder for data collection. Mothers joined the Zoom session from their family home using their own Internet enabled device. Although step-by-step guidance on using Zoom technology was available as needed, all participating parents reported prior experience using Zoom and did not require additional training to use the platform. Parent child observations were conducted during daily family routines selected by the parent in the typical location for these routines. Additional materials included questionnaires distributed via email or Qualtrics. Visual supports (e.g., visual schedules, choice boards, token systems) and timers were mailed to family.

### 2.3 Interventionist and research assistants

The first author, a Chinese woman who is a native Mandarin speaker, served as the PTR-F interventionist for all families who participated in this study. She held a master’s degree in and was a doctoral candidate in special education. She had completed 21 credit hours of approved behavior-analytic coursework and 1,500 hours of concentrated supervised fieldwork towards application for Board Certified Behavior Analyst (BCBA) examination for certification.

Three trained graduate students in special education or ABA served as research assistants to independently code data from video-recorded sessions. Two students were Chinese and fluent in Mandarin and one was Vietnamese.

### 2.4 Dependent variables

#### 2.4.1 Targeted routine and challenging behavior

As noted above, each mother identified topographies of negatively reinforced challenging behavior in the form of escape or avoidance from activities the child experienced as aversive (i.e., homework or Table work, structured play and exercise). Target challenging behavior during a selected family routine was individually defined for each child and the interventionist used the results of the functional behavior assessment (FBA) to operationally define target challenging behavior. Although play was selected as a target routine for some dyads, FBA results confirmed that challenging behavior occurred in the context of structured, adult-directed activities with rules requiring sustained attention and compliance; children engaged appropriately in unstructured, rule-free activities without demonstration of challenging behavior. Challenging behavior was coded for each experimental session according to each child’s individualized operational definitions (see **Table 3**) using 10-s partial interval time sampling. Each 10-min. observation session was split into 60 10-s intervals. Observers independently viewed video recorded sessions and using paper data collection sheets and an audible smartphone-based timer to mark the intervals, selected an interval for target challenging behavior if any of the behaviors occurred during the 10-s. interval. If a target behavior was observed at the auditory cue to mark the end/beginning of an interval, the behavior was coded as occurring in both adjacent intervals. For each experimental session, the percent of time a child exhibited target challenging behavior was calculated by dividing the number of intervals with target challenging behavior by the total number of observed intervals and multiplying by 100 to obtain a percentage which was graphed for analysis.

**Table 3:** Targeted family routine and escape maintained challenging behavior for each child participant.

Participants	Targeted routine	RBI^a^	Target challenging behavior	Operational definition
Jie	Homework	1	Non-compliance	When asked to engage in homework task, verbally protests (e.g., *Bye-bye.; I am done.; No, no, no, I do not like it.*), requests different activity (e.g., *Hug?; Ice cream?; Mommy*)*?*, or engages in non-word protests including rubbing eyes, dropping body to floor, eloping from designated area, crying, screaming, yelling, or whining (low-pitched, nasal sound without words).
Yiyi	Structured Play^b^	2	Non-compliance	When asked to sit on chair to play, verbally protests (i.e., *No.*) or engage in non-word protests including standing still without sitting in chair, eloping from designated area, crying, or whining (low-pitched, nasal sound without words).
Wei	Exercise	3	Non-compliance	When asked to run on treadmill, verbally protests (e.g., *No running.*) requests different activity (e.g., *I want to play, Go upstairs?, Sleep*)*?*, or engages in non-word protests including crying, yelling, running away from the treadmill, or stomping feet.
Lanlan	Structured Play^b^	1	Inattention	During playtime, disengages from task and turns head to look elsewhere, ignores instructions, or elopes from designated area.
Ningfeng	Table work	1	Non-compliance	When asked to participate in table work, engages in non-word protests including eloping, dropping body to the floor, screaming or crying.
Meisheng	Homework	2	Tantrums	When asked to engage in homework task, verbally protests (e.g., No.) or engages in non-word protests (e.g., screams, cries, yells, hits, kicks, throws objects, throws himself onto the floor, stomps feet, stands on and jumps on the flat surface of the desk or chair).

^a^ Routines-based interview (RBI; 26) rating used a scale of 1-5 (1 = terrible; 5 = fantastic).

^b^ Structured play routines consisted of adult-directed, rule-based activities requiring sustained attention and compliance with maternal instructions.

#### 2.4.2 Parents’ treatment fidelity of BSP

Individualized task analyses were created based on each child’s BSP and used to code parent treatment fidelity for each experimental session (see **Supplementary Table 1**). The percent of parents’ treatment fidelity was calculated by dividing the total number of steps correctly completed by the parent by the total number of applicable steps in the child’s BSP and multiplying that number by 100 to obtain a percent of steps completed correctly. Parent treatment fidelity was graphed for each intervention session, and for the baseline phase *post-hoc* data collection after the BSP development indicated families already used some of the strategies chosen as PTR-F strategies.

#### 2.4.3 Procedural fidelity

Parent education and training and coaching protocol checklists were developed to record the implementation of planned steps by the interventionist. These checklists were used by two independent observers while viewing videorecorded sessions to determine procedural fidelity during each relevant session. Procedural fidelity checklists are available as **Supplementary Tables 2, 3**. An overall average of implementation fidelity was calculated by dividing the total number of items marked “Yes” by the total possible items on the checklist and multiplying the answer by 100 to obtain a percentage. The procedure fidelity of training was 100% for all sessions and all participants except for Lanlan’s parent education session. Lanlan’s mother was interrupted and left the session early due to family emergency. She missed the step of retelling the implementation checklist to the interventionist; interventionist training fidelity was 93% for this session. The procedural fidelity of the coaching phase during intervention was 100% for all sessions and all participants.

### 2.5 Experimental design and analysis

This study utilized two independent multiple baseline designs (MBD) across six parent-child dyads (i.e., 3 participants in each MBD) with case randomization and range-bound intervention start point randomization to examine the relation between parent use of a culturally adapted comprehensive BSP developed through the PTR-F process and child challenging behavior. Participants were randomized into two MBD groups: Dyad 1 (Jie and Ling), Dyad 2 (Yiyi and Minyue), and Dyad 3 (Wei and Xiaoxiao) in the first group; Dyad 4 (Lanlan and Ting), Dyad 5 (Ningfeng and Nana), and Dyad 6 (Meisheng and Yuanyuan) in the second. More specifically, Dyad 1 and Dyad 4 were randomly assigned to the first staggered position, Dyad 2 and Dyad 5 to the second, and Dyad 3 and Dyad 6 to the third. Case and intervention start-point randomization were conducted using ExPRT software (27), specifying a minimum of six baseline observations which consistent with the What Works Clearinghouse (WWC) standards requiring a minimum of five data points per phase to meet design standards (28) and a predetermined acceptable interval of two potential intervention start points per tier. For example, the first tier’s acceptable interval was 6–7 sessions, the second tier was 8–9 sessions, and the third tier was 10–11 sessions. The decision to transition from baseline to intervention for each participant was determined *a priori* through randomization procedures and was not contingent on observed changes or trends in baseline data. Within this framework, the ExPRT software randomly selected the actual intervention start points of 6, 9, and 10 sessions for the first, second, and third tiers respectively, each drawn from their respective two-point acceptable intervals, yielding an overall baseline range of 6 to 11 sessions across all tiers. Specifically, actual baseline lengths selected at random were six sessions for Dyad 1 and Dyad 4, nine sessions for Dyad 2 and Dyad 5, and ten sessions for Dyad 3 and Dyad 6. This approach maintained the time-lagged staggered introduction of intervention across participants characteristic of MBDs, and which controls for common threats to internal validity (29). Utilizing randomization techniques increases a single-case study’s internal validity (decreased Type 1 error) and can increase the study’s statistical conclusion validity as well, which permits the calculation of parametric effect size (30).

#### 2.5.1 Interobserver agreement

A second independent observer took data on child challenging behavior and parent treatment fidelity for IOA. For child challenging behavior, IOA data were collected separately for each participant and each experimental phase. Overall, IOA data on child target behaviors were obtained for a mean of 32.22% of baseline sessions (Range = 30-33.33%) and a mean of 41.67% of intervention sessions (Range = 37.5-50%). Specifically, 33.33% of baseline sessions were coded for reliability for Jie, Lanlan, Yiyi and Ningfeng, and 30% of baseline sessions were coded for reliability for Wei and Meisheng. During the intervention phase, IOA data were collected for 50% of sessions for Lanlan and Yiyi and coded for 37.5% of sessions for each of the remaining participants (i.e., Ningfeng, Wei, Meisheng). For parent treatment fidelity, IOA data on parent strategy use were obtained for a mean of 36.75% of sessions (Range = 33-37.5%). For both child and parent target behaviors, IOA was calculated as point-by-point agreement or the percentage of intervals or steps with an agreement for occurrence and non-concurrence ratings (31). It was calculated by dividing the number of agreements between observers by the sum of agreements and disagreements, then multiplying by 100 to obtain a percentage. IOA results are reported in the results section.

### 2.6 Social validity

Following the last intervention session, the researcher asked families to complete two social validity assessments via interview. To measure families’ perceptions regarding the goals, procedures and outcomes of the PTR-F intervention mothers completed a modified version of the social validity measure developed for the randomized controlled trial of PTR-YC (6). The social validity scale includes 10 items answered based on the family’s agreement with a 5-point Likert-type scale. Generally, a score of one indicates low social validity and a score of five indicates high social validity. Additionally, the interventionist modified Fisher and colleagues’ (32, 33) questionnaire to measure mothers’ satisfaction with the telepractice delivery modality. Parents scored each item on a 7-point Likert scale ranging from “1” for “strongly disagree” to “7” for “strongly agree,” with higher scores reflecting greater satisfaction with the rated item.

### 2.7 General procedures

This study consisted of the three phases of pre-baseline assessment, baseline, parent education and intervention, which consisted of interventionist coaching of the parent. Experimental sessions occurred twice weekly, each lasting approximately 30 min. over three to five weeks during the baseline phase and three to four weeks during the intervention phase. Each session was initiated by the interventionist via Zoom session and began with brief greeting and reminder of session agenda.

### 2.8 Pre-baseline assessment

#### 2.8.1 Suinn-Lew Asian self-identity acculturation scale

To assess acculturation levels and to inform culturally responsive adaptations to the intervention, each mother completed the SL-ASIA (34, 35). The SL-ASIA is a 21-item self-report measure assessing dimensions including language use, ethnic identity, friendship patterns, and cultural practices. Items are rated on a 5-point Likert scale, with scores ranging from 1.00 (low acculturation, high Asian identification) to 5.00 (high acculturation, high Western identification). The scale has demonstrated high reliability across various Asian American subgroups (α = .88–.91; 34). Given the first-generation status of the participating mothers, the SL-ASIA (35) results showed uniformly low levels of acculturation (M = 54, Median = 54, Range = 49 - 60). Most mothers preferred speaking Chinese or mostly Chinese; only Minyue reported equal preference for both Chinese and English. All participants read Chinese better than English, and most wrote Chinese more fluently with the exception of Minyue. All identified as Asian/Chinese and primarily associated with Asian peers, although some expressed openness to broader social engagement. There was consistent preference for Asian food, holidays, and cultural values related to family, education, and work.

#### 2.8.2 Functional behavioral assessment

Prior to the baseline phase, an FBA was conducted. According to the PTR-F manual, the FBA process involves three checklists completed by all team members, including any extended family members involved with the child. The interventionist met each participating parent for approximately 1 hr. and 30 min. to support checklist completion and to develop a summary hypothesis statement regarding the perceived communication function of their child’s target challenging behavior. The interventionist also conducted a direct observation of the mother and child in the target routine to confirm the developed summary statements. During these observations, the interventionist took antecedent-behavior-consequence data using an A-B-C data collection form and determined patterns suggesting potential operant functions maintaining the target challenging behavior. Any discrepancies between parent report and interventionist observation were discussed with caregiver, and the summary hypothesis statement revised accordingly. Each child engaged in negatively reinforced escape maintained challenging behavior.

### 2.9 Baseline

Parents were asked to participate in the target family routine with their children as usual, without receiving any feedback or instruction from the interventionist, establishing the baseline. Baseline data collection continued for each dyad based on the number of pre-determined sessions according to the randomly determined rangebound intervention start point.

### 2.10 Parent education and training

#### 2.10.1 Culturally adapted prevent-teach-reinforce for families program

The PTR-F manual provides step-by-step instructions, checklists, templates, and evaluation tools that help interventionists guide caregivers to use. It includes a standardized five-step process: (a) initiating the PTR-F process, during which the intervention team is established, roles are clarified, and meaningful goals, routines, and target behaviors are identified; (b) PTR-F assessment, which involves a routine-based functional behavioral assessment to understand the environmental conditions maintaining challenging behavior; (c) PTR-F intervention, in which an individualized BSP incorporating prevent, teach, and reinforce strategies is developed based on assessment results; (d) coaching, which supports parents’ accurate and sustained implementation of the BSP through ongoing guidance and feedback; and (e) monitoring plan implementation and child progress, which involves tracking fidelity and child outcomes to inform data-based decision making. This standardized PTR-F framework served as the foundation for intervention delivery, with culturally responsive adaptations applied to specific components.

The adaptations to the PTR-F program were made by using the EVM of Bernal et al. (20). First, prior to BSP development, adaptation decisions were informed by multiple data sources, including: (1) screening data (e.g., acculturation level and language preference), (2) pre-baseline interviews with parents regarding their child’s diagnostic history and family perceptions of disability, and (3) direct observations of parent–child interactions within daily family routines. Second, these data were synthesized by the interventionist to identify culturally salient values, communication preferences, family dynamics, and contextual factors with potential implications for intervention acceptability and implementation. Third, identified cultural considerations were systematically mapped onto the EVM dimensions to guide adaptations to intervention language, instructional materials, delivery roles, goal selection, and contextual emphasis.

##### 2.10.1.1 Language

Studies have found that language modification increases acceptance of intervention content, supports higher retention rates, and increases the likelihood of treatment uptake (36, 37). To address language barriers, the interventionist emphasized her Chinese cultural and language background during recruitment and administered the SL-ASIA at first contact to assess each mother’s acculturation level and language preference. Based on SL-ASIA results indicating low acculturation across all six mothers (scores ranging from 49–60 out of 130), all experimental sessions were delivered in Mandarin. Key materials in the PTR-F family manual and all study questionnaires were translated into Mandarin, with both English and Mandarin versions provided to all families.

##### 2.10.1.2 Person

The person dimension of the EVM refers to the match between client and interventionist in terms of cultural background, values, and relational expectations. In Chinese culture, professionals are typically regarded as authority figures (38). Participating mothers tended to rely on the interventionist for decision-making throughout the process. To address this dynamic, the intervention was adapted so that the interventionist served as a culturally matched expert authority figure who invited parent participation without expecting mothers to lead the process. The interventionist presented multiple BSP strategy options for parents to consider and guided mothers in selecting strategies that aligned with both the child’s needs and the family’s cultural preferences. Additionally, the interventionist conducted direct observations to verify FBA hypothesis statements and took detailed notes during baseline observations to support BSP development, reducing the burden on mothers to independently generate intervention strategies. Recruitment was facilitated through a WeChat group of Chinese American parents of children with developmental disabilities, and all participating mothers cited the interventionist’s shared Chinese cultural background as a primary motivation for enrollment.

##### 2.10.1.3 Metaphors

Culturally specific proverbs, sayings, and idioms (谚语/俗语和成语) are deeply embedded in Chinese communication and can anchor abstract intervention concepts in familiar cultural knowledge, increasing accessibility and acceptance of intervention content (20). The interventionist incorporated Chinese proverbs and idioms into PTR-F manual materials and parent discussion sessions to explain key intervention concepts. For example, 三位一体,一环扣一环 (Three in One, One link to another) was used to explain the relationship between Prevent, Teach, and Reinforce; 父母是孩子最好的老师 (Parents are the child’s best teachers) was used to emphasize parents’ unique role in supporting their children’s development; and 万事皆有因果 (Everything has a cause and consequence) was used to illustrate the cause-and-effect framework underlying FBA. Additional proverbs were used to explain core ABA principles, including 防患于未然 for the prevention principle, 熟能生巧 for repetition and precision teaching, and 习惯成自然 for the maintenance principle.

##### 2.10.1.4 Content and concepts

Cultural context shapes how caregivers understand and respond to their child’s disability diagnosis and challenging behavior, which in turn affects engagement with intervention content (39). Chinese parents of children with ASD commonly lack knowledge about ASD diagnosis and etiology, report high levels of self-blame, and experience stigma associated with having a child with a disability, as any misbehavior of the child is often attributed to parental failure in Chinese culture (40, 41). To address these cultural factors, the interventionist conducted a structured interview with each mother prior to parent education to understand her child’s diagnostic history and the family’s perceptions of disability and challenging behavior. This interview informed the interventionist’s approach to parent education and revealed significant variation across families. For example, Minyue initially attributed her son’s autism to environmental causes and sought a second diagnosis in China before accepting the diagnosis, while Nana believed her son would eventually recover, viewing autism as an illness rather than a neurodevelopmental disability. Based on these findings, the interventionist added psychoeducational components about ASD diagnosis, etiology, and the relationship between ASD and challenging behavior to the standard PTR-F parent education materials, which typically focus exclusively on intervention strategies. Additionally, a direct observation procedure was added to the FBA phase to verify hypothesis statements developed collaboratively with parents.

##### 2.10.1.5 Goals

Chinese culture’s emphasis on collectivism, family harmony, and preserving face (面子) in the community shapes how caregivers define and prioritize behavioral goals for their children (42). Participating mothers in this study characterized their children’s challenging behavior primarily as disobedience and prioritized goals reflecting family cohesion, respect for authority, and reduction of public embarrassment. The interventionist supported mothers in identifying and selecting target behaviors aligned with these cultural values. For example, Ningfeng and Meisheng’s mothers chose following maternal instructions as the primary target behavior, directly linked to concerns about losing face when their children exhibited challenging behaviors in public. Many mothers also reported receiving frequent negative feedback from their children’s classroom teachers, contributing to feelings of shame; as a result, Jie, Ningfeng, and Meisheng’s mothers initiated homework and academic activities at home as target routines, motivated by a desire to reduce teacher complaints and improve their children’s school behavior.

##### 2.10.1.6 Methods

WeChat, a widely adopted bilingual messaging platform among Chinese immigrants in the U.S. with approximately 70 million overseas monthly active users (43), was used as the primary communication tool throughout the study for scheduling sessions, sending reminders, and addressing questions between sessions. The use of a culturally familiar communication platform supported relationship building between the interventionist and participating families, consistent with Chinese cultural emphasis on long-term client-provider relationships (44). Additionally, the entire intervention was delivered via telepractice to increase access for Chinese American families across the U.S. who lack access to culturally and linguistically matched behavior specialists, a significant barrier to service access for this population (10, 14).

##### 2.10.1.7 Context

The context dimension of the EVM addresses the social, familial, and environmental factors that shape intervention delivery and acceptability. Chinese American families may experience unique contextual stressors including acculturative stress, social isolation, and complex extended family dynamics related to child-rearing expectations and family lineage (38). Several participating mothers reported significant stress in their relationships with spouses and mothers-in-law, who at times attributed the child’s challenging behavior to the mother’s inadequate caregiving. These dynamics directly informed routine selection and session delivery. For example, Ningfeng’s target routine was selected without involving his father due to marital conflict; when his father was home, the interventionist allowed Ningfeng’s mother to move to a separate room to receive performance feedback more comfortably. The interventionist consistently provided emotional support and positive reinforcement to address caregiver burden, acknowledged mothers’ strengths and resilience, and connected families to local support networks and online communities.

Components of the cultural adaptation and examples are summarized in **Table 4**.

**Table 4:** Cultural adaptation components and examples of planned adaptations of PTR-F program for participating parents.

Cultural dimension (EVM)	Cultural finding	Adaptation strategy	Implementation examples
Language	• Participants demonstrated limited English proficiency and low acculturation levels (SL-ASIA scores 49–60/130) • All six mothers preferred Mandarin for all services	• Provide linguistically accessible materials and instruction to support treatment uptake and retention.	• The interventionist translated the recruitment flyer and emphasized the interventionist’s Chinese culture and language background when recruiting parents to PTR-F • Translation of researcher created study materials into Mandarin, key materials in PTR-F manual translated into Chinese, but all parents received print materials in both English and Mandarin • Mothers completed SL-ASIA at first contact to inform comfort level and confidence in communicating in English and Mandarin • Based on participating mothers’ input, all experimental sessions delivered in Mandarin
Person	• In Chinese culture, professionals are typically regarded as authority figures • Participating mothers tended to defer to the interventionist for decision-making rather than leading the collaborative process • All mothers reported the interventionist’s shared Chinese cultural background as a key motivating factor for participation	• Bilingual Chinese interventionist served as a culturally matched expert authority figure • Intervention model adapted to invite parent participation without expecting mothers to lead	• Interventionist presented multiple BSP strategy options and guided mothers in selecting strategies aligned with child needs, family preferences, and cultural context • Interventionist conducted direct observations to verify FBA hypothesis statements and took notes during baseline to support BSP development.
Metaphors	• Culturally specific proverbs or sayings and idioms increase parent access to the intervention by anchoring meaning in prior knowledge	• When creating materials and discussion sessions with parents, the interventionist incorporated culturally specific proverbs/sayings and idioms (谚语/俗语和成语 in Chinese)	• 三位一体,一环扣一环 (Three in One, One link to another) to explain the relationship between “Prevent, Teach, Reinforce” • 父母是孩子最好的老师 (Parents are the child’s best teachers in the world) to emphasize the idea that parents are uniquely positioned to provide their children with the guidance, support, and education they need to thrive • 万事皆有因果 (everything has a cause and consequence), to illustrate the cause-and-effect relationship between a behavior and its antecedents and consequences
Content and Concepts	• Chinese parents of children with ASD commonly lack knowledge about ASD diagnosis and etiology, report high self-blame, and experience stigma associated with having a child with a disability • In Chinese culture, child misbehavior is often attributed to parental failure • Participating mothers varied widely in their understanding of ASD etiology and the relationship between ASD and challenging behavior, with some holding misconceptions that required direct address	• The interventionist conducted a short interview with parents before parent education to understand their children’s diagnosis process and their family’s perceptions about their child’s disabilities and challenging behavior • The interventionist included psychoeducational components about ASD diagnosis in parent education materials typically only focused on intervention strategies	• Minyue initially suspected autism was caused by environmental pollution (proximity to a river) and sought a second diagnosis in China before accepting the diagnosis • Nana viewed autism as an illness rather than a neurodevelopmental disability • Ling saved her younger daughter’s cord blood hoping to treat autism
Goals	• Chinese culture emphasizes collectivism, family harmony, and preserving face (面子) in the community • Participating mothers characterized their children’s challenging behavior primarily as disobedience rather than disability-related behavior • Some mothers expressed heightened concern about children’s behavior in public settings and negative feedback from classroom teachers, contributing to feelings of shame • Some family strong emphasis on respecting authority figures including teachers	• Interventionist supported mothers in selecting target behaviors reflecting rule- following, compliance with adult instructions, and appropriate behavior in structured settings, rather than imposing goals oriented toward individual child autonomy	• Several mothers characterized their child’s behavioral issues as disobedience and wanted their child to better follow instructions (e.g., Meisheng and Ningfeng’s mothers expressed feelings of losing face and shame when their children exhibited challenging behaviors in public. They each chose following instructions as a desired intervention outcome) • Many parents reported receiving frequency negative feedback from their child’s classroom teacher, which contributed to feelings of shame and a sense of having caused trouble for the teachers. Parents were motivated to address their child’s behavior at home and improve the school situation (e.g., Jie, Ningfeng, and Meisheng’s mother started homework or some academic activities at home)
Methods	• Bilingual technology (Mandarin and English) can assist in reducing language barriers and fostering trust by aligning with participants’ communication preferences • Step-by-step instructions that align with traditional educational expectations	• WeChat was used by the interventionist to facilitate communication with participants, including scheduling sessions, sending reminders, and answering questions • Intervention delivered entirely via telepractice to increase access to underserved families across the U.S. who lack access to culturally and linguistically matched providers • Used standardized checklists from the PTR-F manual but adapted them into simplified, bilingual visual schedules for parents to follow during daily routines	• All six mothers communicated with the interventionist exclusively through WeChat throughout the study
Context	• The service delivery model, and understanding relevant social elements such as acculturative stress, social support, family relations, and parent preference are relevant to providing behavioral consultation with cultural humility	• This study emphasized the daily routines of the participating families since this can differ between Chinese American families and families of mainstream American culture due to variations in cultural values, priorities, and daily practices • Several mothers reported relationship stress with spouses and extended family, including pressure from mothers-in-law and expectations regarding child- rearing and family lineage	• For instance, Ningfeng’s mother expressed fear of being accused by her husband of inadequately caring for and educating Ningfeng at home. As a result, Ningfeng’s routine was selected by his mother alone. If his father was home, the interventionist allowed Ningfeng’s mother to go to another room where she felt more comfortable receiving performance feedback

#### 2.10.2 Behavior support plan development

To develop BSP, the interventionist met each family for approximately 1 hr. 30 min. to introduce the PTR-F program’s four universal practices, twelve prevent strategies, six teach strategies, and three reinforce strategies. Guided by the FBA and baseline observations, the interventionist provided professional recommendations, from which parents selected at least one prevent, one teach, and one reinforce strategy targeting the function of their child’s challenging behavior. Consistent with the cultural adaptation to the *Person* dimension of the EVM, participating mothers initially selected as many strategies as possible, expressing a preference for professional guidance over independent decision-making. Mothers indicated limited familiarity with ABA-based strategies and deferred to the interventionist’s recommendations, reflecting the cultural norm of professional deference common among Chinese immigrant families. The interventionist provided professional suggestions and multiple options based on FBA results and baseline observations, and mothers were more likely to select interventionist-recommended strategies than to independently identify their preferred strategies.

In addition, strategy selection and implementation were further informed by cultural considerations identified through the EVM adaptation process. For example, “Teach Social Skills – Following rules” was frequently selected across families, reflecting parental emphasis on obedience, rule compliance, and maintaining harmony within structured routines such as homework time. Similarly, strategies such as “Change How Instructions Are Delivered” and “Modify What Is Explicitly Asked of the Child” were implemented in a manner that preserved parental authority while reducing escalation. Reinforcement procedures emphasized frequent verbal praise for small incremental improvements, representing a culturally responsive shift toward increasing positive parent–child interaction patterns. Participating mothers were coached to provide immediate and generous praise for demonstrations of rule-following or task engagement. This emphasis reflected that praise was used relatively sparingly in these families, consistent with Chinese parenting norms that frequent praise may be perceived as undermining modesty or intrinsic motivation (45), which may differ from the high rates of positive reinforcement typically emphasized in ABA-based intervention. BSPs incorporated family-identified goals, specified strategy implementation steps, and outlined required materials. Each participant’s BSP strategies are shown in **Table 5**.

**Table 5:** Strategies on the behavioral support plan.

Participant	Prevent	Teach	Reinforce*
Jie	Enhancing Predictability with Schedules Use Timers and Other Visual or Auditory Supports for Added Information or Structure Remove Triggers for Challenging Behaviors Provide a Warning to Inform the Child of Follow-Up Activities	Teach Social Skills – Following rules Teach Independence with Visual Schedules and Calendars	Focus on Jie’s appropriate behavior during the homework time
Yiyi	Enhancing Predictability with Schedules Use Timers Provide choice Provide a Warning to Inform the Child of Follow-Up Activities Modify What Is Explicitly Asked of the Child	Teach Social Skills – Following rules Teach Appropriate Ways to Communicate	Focus on Yiyi’s appropriate behavior during the homework time, and always praise the appropriate behavior with verbal praise, M&M chocolate.
Wei	Use Timers Provide choice Remove Trigger Provide a Warning to Inform the Child of Follow-Up Activities	Teach Social Skills – Following rules	Focus on Wei’s appropriate behavior during the exercise time, and always praise the appropriate behavior with thumbs up, verbal praise. If you cannot ignore challenging behavior, use neutral prompt back to schedule. Reinforce re-engagement quickly.
Lanlan	Provide choice Use Timers Provide a Warning to Inform the Child of Follow-Up Activities Reduce Distractions and Materials Modify What Is Explicitly Asked of the Child Change How Instructions Are Delivered	Teach Appropriate Ways to Communicate	Focus on Lanlan’s appropriate behavior during the Play time, and always praise the appropriate behavior with verbal praise. Mom did not provide any complain/negative comments to Lanlan’s behavior
Ningfeng	Provide choice Enhancing Predictability with Schedules Provide a Warning to Inform the Child of Follow-Up Activities Remove Triggers for Challenging Behaviors Reduce Distractions and Materials Change How Instructions Are Delivered	Teach Social Skills – Following rules Teach Independence with Visual Schedules and Calendars	Focus on Ningfeng’s appropriate behavior during the homework time, and always praise the appropriate behavior with verbal praise. When Ningfeng is leaving, mom does not follow him, just stay in the space, and keep prompt him to come back.
Meisheng	Enhancing Predictability with Schedules Remove Triggers for Challenging Behaviors	Teach Social Skills – Following rules Teach Independence with Visual Schedules and Calendars	When he engaged tantrum behavior, mom does not provide any option to him.

*All reinforce plans must: (1) Identify a functional reinforcer (s), (2) Provide reinforcer for desirable behavior, (3) Remove reinforcement for challenging behavior.

#### 2.10.3 Parent education and training

Each parent and any additional family members nominated by the mother participated in two one-hour parent education sessions focused on implementing the selected BSP strategies. Parent training followed a structured instructional approach consistent with behavioral skills training (BST), including instruction, modeling, rehearsal, and performance feedback. The sessions began with a structured review of selected strategies from the categories of prevent, teach, and reinforcement, accompanied by rationale and examples of how to apply them. For instance, Ling was guided to use a visual schedule and timer to help Jie transition from snack time to homework. She was instructed to show Jie his afterschool schedule, inform him of the next activity (e.g., “Homework time in 3 minutes”), and to remind him of remaining time before the transition. Parents were provided with an individualized treatment fidelity checklist for each BSP strategy (see **Supplementary Table 1**). Parents were encouraged to ask questions. Next, the interventionist modeled each strategy step-by-step, demonstrating correct implementation while highlighting potential challenges and addressing parental questions (e.g., interventionist modeled how to use specific verbal cues with the timer [“Jie, you have 10 more minutes for a snack, then homework”] and discussed how to address refusal to transition). To assess understanding, the interventionist: (1) asked scenario-based questions (e.g., “How to help Jie transition from snack time to homework time? What should you do when Jie starts to whimper or ask for a hug?”); (2) asked parents to verbally describe strategy steps using the checklist, and (3) reviewed baseline videos with reflective prompts (e.g., “What would you do differently here?”). Each rehearsal was followed by feedback, with the interventionist highlighting correct actions, correcting errors (e.g., when Ling omitted the phrase “then homework”), and offering additional practice opportunities. Fidelity of the parent training procedures was monitored using a procedural fidelity checklist (see **Supplementary Table 2**). Training concluded when parents demonstrated at least 80% accuracy in reciting strategy steps using the checklist.

#### 2.10.4 Behavior support plan implementation and performance feedback

Following the parent education session, mothers were asked to implement the BSP during the selected routine. During parent-mediated intervention, the interventionist used a fidelity checklist to score implementation and take notes. Immediately after implementation, the mother briefly exited the Zoom meeting while the interventionist exported the session video to facilitate discussion and interventionist delivery of performance feedback to support mothers’ implementation of their child’s BSP. The interventionist first prompted parent reflection using questions such as, “How did you feel?”, “What was difficult?” After addressing parent responses, structured feedback was provided using a systematic framework (Shuler & Carroll, 2019): (a) praise for accurate implementation, (b) identification of errors, (c) rationale for corrections, (d) guidance for correct performance, (e) demonstration of correct behavior, (f) opportunities to rehearse correct behavior if needed, and (g) time for questions. Each session concluded with a summary and reminders for key points to focus on in the next session. Coaching sessions continued until mothers achieved ≥80% fidelity for three consecutive sessions. The total dosage of performance feedback and coaching across parents averaged 181 minutes (approximately 3 hours), with a range of 93 to 296 minutes.

## 3 Results

**Figure 1:**
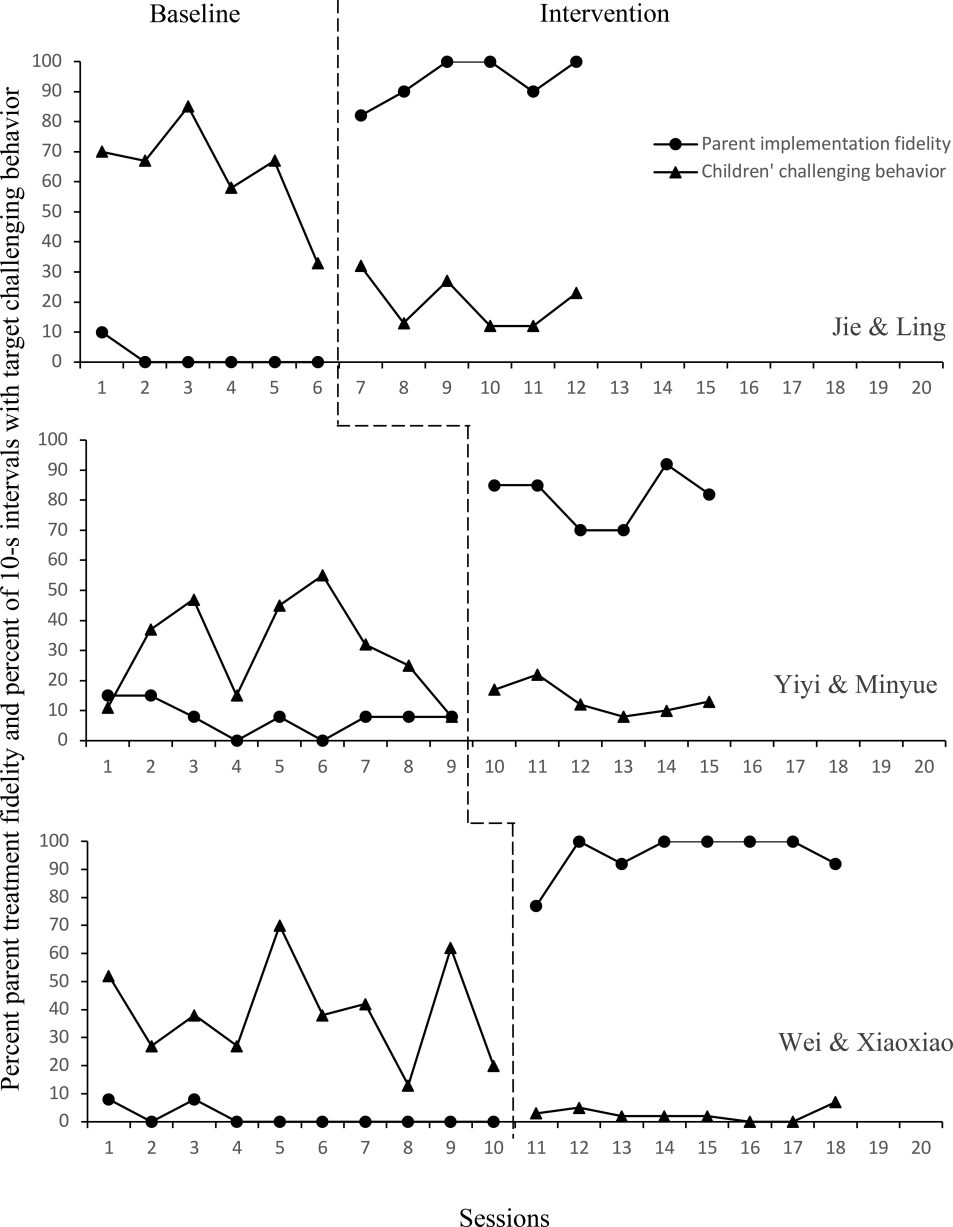
The relation between parents’ fidelity to the implementation of BSP strategies and children’s challenging behavior in the first MBD group.

**Figure 2:**
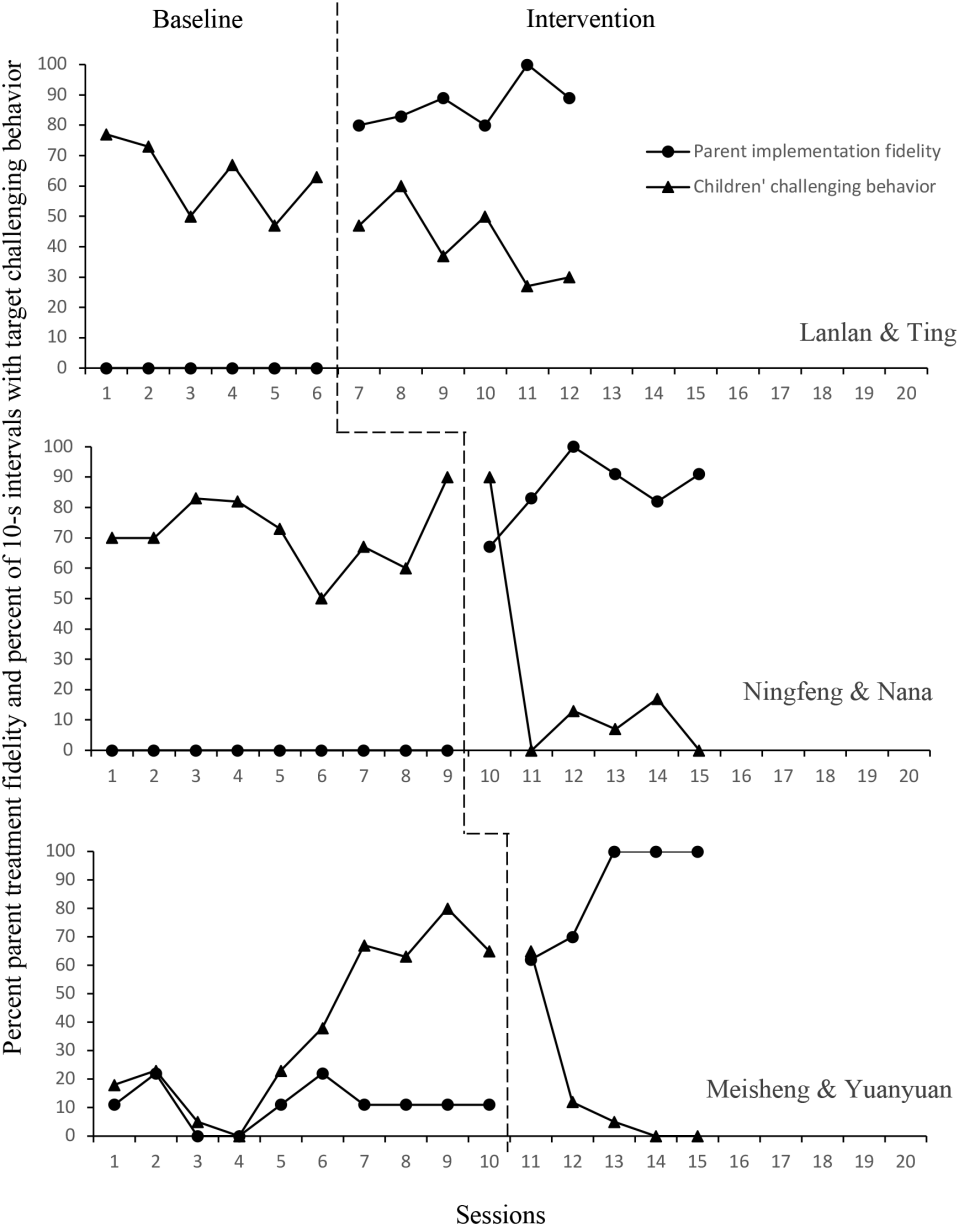
The relation between parents’ fidelity to the implementation of BSP strategies and children’s challenging behavior in the second MBD group.

**Figures 1**, **2** display the results of each independent randomized multiple baseline designs in baseline and intervention.

### 3.1 Parents’ fidelity to implementation of BSP

Retrospective baseline data collection demonstrated four of the six mothers implemented some of the PTR-F strategies eventually selected for the BSP. Specifically, both Ling and Xiaoxiao used timers, Minyue used choice making, and Yuanyuan attempted to use several strategies (i.e., timer, visual schedule, cards) prior to intervention. Nevertheless, baseline strategy use remained low for these mothers (M = 4.33%; range = 0% - 22% Following parent education and coaching, each mother immediately improved in the level of their treatment fidelity and achieved 80% fidelity (M = 87.83%; range = 62% - 100%) for at least 3 consecutive sessions.

### 3.2 Children’s challenging behavior

Jie. During baseline, Jie displayed high levels of challenging behavior, with no clear trend but high variability (M = 63%; range = 33% - 85%) in the baseline. For his last baseline session, Jie engaged in challenging behavior for 33% of intervals, which was well below previous sessions. Although this low score was attributed to Jie’s illness on that day, it is likely that his mother also reduced the frequency and intensity of task demands in response to his illness, resulting in fewer antecedent triggers for escape-maintained challenging behavior rather than reflecting a true behavioral change. Upon implementation of BSP, data were stable (M = 20%; range = 12% - 32%) with an immediate decreased level and trend of Jie’s challenging behavior.

Yiyi. Yiyi’s challenging behavior showed some fluctuations but an overall higher level, with a moderate variability (M = 31%; range = 8% - 55%) in the baseline phase and a decreasing trend prior to intervention. Upon implementation of the BSP phase, no immediate change in level was observed. The data were stable (M = 14%; range = 8% - 22%) with a slight decreasing trend and low level.

Wei. Wei displayed high levels of challenging behavior and high variability (M = 38.9%; range = 13% - 70%) in the baseline with a decreasing trend prior to intervention. Upon implementation of the BSP phase, an immediate change was observed. A gradual decreasing trend, minimal variability, and low levels of challenging behavior were observed (M = 2.6%; range = 0% - 7%).

Lanlan. Lanlan displayed high levels of challenging behavior, with a slight decreasing trend and moderate variability with fluctuations (M = 62.83%; range = 47% - 77%) throughout the baseline. Upon implementation of the BSP phase, it still showed a decreasing trend, but an immediate change was observed. Challenging behavior did change in level and variability (M = 42%; range = 27% - 60%).

Ningfeng. Ningfeng’s baseline data showed a relatively stable but high level of challenging behavior (M = 74.44%; range = 50% - 90%). Upon implementation of the BSP phase, no immediate change was observed. There was one data overlap between baseline and intervention conditions. High variability in the intervention scores, with a dramatic decrease followed by low scores (M = 21%; range = 0% - 90%) were observed.

Meisheng. Meisheng displayed a low to moderate level of challenging behavior with a fluctuating trend and high variability (M = 38.7%; range = 0% - 80%) during the baseline phase. Upon implementation of the BSP phase, no immediate change was observed. A downward trend was observed after the first intervention score. There was moderate variability in intervention scores (M = 16%; range = 0% - 65%). His mother Yuanyuan changed the schedule from the sixth session, and then his challenging behavior increased from the sixth session to 42%, 58% for the seventh session, and 63% for the eighth session. For example, he only was required to read one book under the reading schedule, but his mother changed to read two books. Meisheng engaged in hitting to protest the additional demand.

### 3.3 Interobserver agreement

IOA on child challenging behavior across baseline and intervention for each child showed that Jie’s refusal behavior averaged 87% (range 83–90%) in baseline and 96% (range 93–97%) in intervention, Yiyi’s refusal behavior averaged 92% (range 87–97%) in baseline and 87% (range 80–93%) in intervention, Wei’s refusal behavior averaged 95% (range 93–100%) in baseline and 97% (range 90–100%) in intervention, Lanlan’s inattention behavior averaged 81% (range 77–90%) in baseline and 86.5% (range 80–93%) in intervention, Ningfeng’s escape behavior averaged 93% (range 87–100%) in baseline and 97% (range 90–100%) in intervention, and Meisheng’s tantrum behavior averaged 94% (range 87–100%) in baseline and 100% in intervention. IOA for parent treatment fidelity averaged 97% for Ling (range 91-100%), 88% for Minyue (72-100%), 100% for Xiaoxiao, 89% for Ting (78-100%), 100% for Nana, and 100% for Yuanyuan. IOA data on procedural fidelity were collected for 50% of the training sessions and the resultant IOA was 100%.

### 3.4 Statistical analysis

To supplement visual analysis of the graphed data, a Tau-U calculator available at www.singlecaseresearch.org was used to calculate the nonoverlap statistic (46). The data of Jie and Wei in the first MBD group had no overlap, which results in a Tau score of 1 suggesting a large effect of the intervention. The other participants had Tau scores ranging from 0.5 - 0.8, indicating a moderate to large effect of the intervention. Additionally, to determine the treatment effect size of the PTR-F on child target challenging behavior at the study level, the Hedges’ g effect sizes (Hedges, 1981) were computed in SPSS using the DHPS macro (47). In the first MBD, Hedges’ g = -1.58 indicated a very large effect size. For the second MBD, Hedges’ g = -1.14 reflected a large effect size.

### 3.5 Social validity

All six mothers favorably rated the PTR-F process and the BSPs they developed and implemented. **Table 6** shows the social validity ratings for each family across all items on the social validity questionnaire. The mean scores for individual items ranged from 1.0 to 5.0, indicating strong agreement with the statements related to the training experience. In addition, each mother expressed high levels of satisfaction with the virtual training program (see **Supplementary Table 4**). The mean scores for individual items ranged from 6.67 to 7, indicating strong agreement with the statements related to the training experience. The total satisfaction scores ranged from 52 to 56 across families, with an overall mean score of 55.17. These results indicate the families were generally very satisfied with the virtual training program and found it an effective alternative to in-person training.

**Table 6:** Social validity ratings across questions and participating mothers.

Item	Ling	Minyue	Xiaoxiao	Ting	Nana	Yuanyuan	Mean
Acceptability of the PTR Plan	5	5	5	5	5	5	5
Family’s Willingness to Carry Out Plan	5	5	5	5	5	5	5
*Disadvantages in Following the Behavior Plan	2	2	1	1	1	1	1.2
* How Disruptive is it to Carry Out the Plan	1	1	1	1	1	1	1
How Much Do You Like the Proposed Plan Procedures	5	5	5	4	5	5	4.8
* Extent that Undesirable Side- Effects Result from the Behavior Plan	1	1	1	1	1	1	1
* How Much Discomfort is Your Child Likely to Experience During the Behavior Plan	1	2	2	2	1	1	1.5
How Well Does the Behavior Plan Fit into Existing Routine	5	5	5	5	5	5	5
Effectiveness of Plan in Teaching Child Appropriate Behavior	5	5	5	4	5	5	5
Fit of Plan with Family’s Goals to Improve Child’s Behavior	5	5	5	4	5	5	5
Comments	希望这个项目能够推广, 让更多的家庭受惠. (*I hope this project can be promoted so that more families can benefit from it*)	*I like this program, especially the visual scheduling and the time to make it easy to transit my son from one activity to another. I feel more comfortable to engage my son to some educational games after this program* Very appropriate.”	*I have applied the method to mealtime routine, and it is working well*	*It will be great if the researcher can give some instruction of what and how they want the parent to play with the kids before starting the observing session*	非常感谢, 很好帮助, 效果很好. (*Thanks a lot, very helpful, works great for my child and my family*)	对孩子帮助很大 (*It’s very helpful for my child*)	
Total score	35	36	35	32	34	34	34

Generally, a score of one indicates low social validity and a score of five indicates high social validity. Questions marked with an asterisk were rated inversely, with a score of 1 indicating the most favorable rating and a score of 5 indicating the least favorable rating.

### 3.6 Summary

Despite strong effect size estimates for intervention effects at the individual participant and study level and high social validity from parent report, the findings are somewhat mixed in our confidence in the functional relation between the intervention and decreased challenging behavior. A strong, indisputable functional relation was demonstrated between the intervention and increased BSP strategy use for all mothers. A strong functional relation was demonstrated between increased BSP strategy use and decreased child challenging behavior for two of the six Dyads and weaker but present participant level effects for Dyads 1,2,3, and 4 due to decreasing baseline trends in challenging behavior and lack of clinically significant decreased challenging behavior for Dyad 4. These findings suggest some caution in interpreting the effect sizes related to challenging behavior reduction in particular.

## 4 Discussion

This SCED study evaluated the overall effectiveness of a culturally adapted PTR-F program delivered via telepractice to six Chinese American families of young children with ASD. Findings revealed each mother improved in their BSP strategy use following parent education and a strong functional relation was found between parents’ fidelity to the implementation of BSP strategies and decreased child challenging behavior for two of the six dyads. For the children (Dyads 1,2,3,4), decreasing trends of challenging behavior in baseline just prior to intervention decreases confidence in inference of the basic effect of the intervention for these children. Dyad 1 low challenging behavior score in the final baseline session was attributed to illness rather than a systematic behavioral change, as all other baseline sessions showed consistently high levels. On that day, Jie’s mother also reduced the frequency and intensity of task demands, thereby decreasing antecedent triggers for his escape-maintained challenging behavior. This contextual explanation is consistent with the FBA hypothesis that Jie’s challenging behavior was functionally related to task demands rather than reflecting a true behavioral change. Nevertheless, this data point may have attenuated the apparent magnitude of the intervention effect by lowering the baseline mean and reducing the visual contrast between phases. Similarly, intervention data for Dyad 4 suggests a less clinically significant effect.

Consistent with prior studies on PTR-F (10, 11, 14), this study demonstrated parents were able to implement the PTR-F interventions with high levels of fidelity, leading to a reduction in challenging behaviors of children with ASD, and it extended the literature by being the first to culturally adapt PTR-F for Chinese American families.

Social validity findings further corroborate prior research indicating strong family satisfaction with PTR-F (10, 11, 14). Parents perceived the PTR-F program as beneficial to adults as it was to their children. Particularly, Xiaoxiao was pleased to see the significant changes in Wei’s behavior during mealtimes, which was not a targeted routine in this study, supporting earlier evidence of PTR-F’s generalization effects (12). In alignment with prior PTR-F studies utilizing telepractice (10, 14), this study also found delivering PTR-F remotely was feasible and well-received by families. Parents consistently reported high levels of satisfaction with the telepractice format, citing convenience, accessibility, and effectiveness. The telepractice delivery model was particularly valuable for families who lacked access to culturally and linguistically matched providers. For example, Ting shared that prior to this study, she had been unable to access behavior support in Mandarin for her daughter due to the lack of Mandarin-fluent board behavior specialists in her area. These findings underscore the potential of telepractice not only as a viable alternative to in-person services, but also as a critical pathway to increase equity and access for underserved communities. This aligns with prior literature on the effectiveness and social validity of technology-based service delivery in behavioral interventions (e.g., 48–50). Finally, this study found participation in the culturally adapted PTR-F program, which included parent education, training, and coaching, effectively enhanced parental confidence. This finding aligns with research demonstrating parent-mediated programs can also improve caregiver confidence and self-efficacy (5, 51).

This study provides preliminary evidence supporting the feasibility and effectiveness of a culturally adapted PTR-F intervention delivered via telepractice to Chinese American families of young children with ASD. Findings demonstrated parents implemented BSP strategies with high fidelity, resulting in reductions in children’s challenging behavior, while reporting high satisfaction with the intervention and its telepractice delivery format and improved confidence in supporting their child with ASD. This study addresses a significant gap in the literature by advancing culturally responsive and accessible behavioral interventions for Chinese American families. However, although the cultural adaptation process in this study extended beyond linguistic translation to include culturally familiar symbols, values-based framing, and contextual considerations, it represents an initial step, further research is needed to more deeply explore how culturally grounded values, norms, and family practices shape parents’ interpretation and implementation of intervention strategies. Future studies should employ consumer-driven methods to systematically examine these deeper cultural processes and their influence on intervention fidelity, sustainability, and outcomes. Additionally, the use of randomization designs with *a priori* set intervention starts points prevented additional data collection during baseline which could have provided more confidence in the intervention effect by ensuring stability in baseline data prior to intervention. Importantly, four of the six dyads in this study demonstrated decreasing trends in baseline target challenging behavior at the time intervention was introduced, which suggests the potential that an extraneous variable influenced child challenging behavior. However, the use of a randomization single-case design provides an additional layer of credibility to our inference that the intervention contributed to further reduction in child challenging behavior. Randomization approaches have been increasing utilized in single case research over the past 12 years (52). One of these approaches, intervention start point randomization, does deviate from a traditional response guided approach to single-case research. Although there is a risk of problematic trend in baseline such as what was observed in this study, intervention start point randomization does (a) decrease potential researcher bias related to when participants begin intervention; and decrease threats to internal validity including (b) ambiguous temporal precedence, maturation, and regression to the mean (29). In this study, a methodological trade off was made to use this approach rather than a traditional response guided approach so that we could decrease Type 1 error and improve statistical conclusion validity. Furthermore, although the PTR-F program was systematically adapted in this study to address cultural and linguistic factors relevant to Chinese American families, this study did not directly examine parents’ perceptions of the specific cultural adaptation components. It is unknown whether families found certain adaptations—such as the inclusion of cultural metaphors, language matching, or values-based framing—particularly helpful or less relevant. Without this assessment, it is challenging to determine whether the adaptations were perceived as relevant and effective by the parents. Future research should continue refining adaptation processes to further enhance the contextual and cultural fit of parent-mediated behavioral intervention for Chinese American families.

## Data Availability

The original contributions presented in the study are included in the article/**supplementary material**. Further inquiries can be directed to the corresponding authors.
