# Supplementary material for: Remote delivery of culturally adapted prevent-teach-reinforce for families with Chinese American families of young autistic children
Source: Front Psychiatry. 2026 Apr 21;17:1783825. doi: 10.3389/fpsyt.2026.1783825 (PMC13140853; doi:10.3389/fpsyt.2026.1783825)
Supplement: Supplementary file 1 [file SupplementaryFile1.zip › Supplementary Table 1 Individualized treatment fidelity checklists across dyads.docx]

**Supplementary Table 1** Individualized treatment fidelity checklists across dyads

**Dyad 1** Jie and Ling

| **PTR-F Fidelity of Strategy Implementation Form** | |
| --- | --- |
| Child: Jie  Date: | Routine: Homework  Person implementing: Mom |
| Strategy steps | Were the steps implemented as intended? |
| Prevent strategy: |  |
| 1. Timer and visual schedule for transition:  - 5 minutes before it is time for Jie to begin his homework routine, Mom will make sure to have Jie’s attention and tell him “5 more minutes until homework. I’m going to set the timer.” Briefly review the daily visual schedule with Jie and set the timer. The timer will be kept out of Jie’s reach. |  Yes  No   Yes  No |
| - 3 minutes before it is time for Jie to begin his homework routine, Mom will make sure to have Jie’s attention and tell him “3 more minute for break, and then homework time.” Briefly review the visual schedule again with Jie and show Jie the timer. The timer will be kept out of Jie’s reach. During this time, also prompt Jie to go to restroom before - homework routine. |  Yes  No |
| - 1 minutes before it is time for Jie to begin his homework routine, Mom will make sure to have Jie’s attention and tell him “1 more minute for break, and then homework time.” Briefly review the visual schedule again with Jie and show Jie the timer. The timer will be kept out of Jie’s reach. - During this time, also show the rule of homework, and the first rule is sat down at the study desk and chair set, and then prompt Jie to come to the worktable and sit down. |  Yes  No   Yes  No |
| - When the timers up and the timer is beeping, Mom will take the timer to Jie and ask, “What time is it? It’s time to get ready for homework.” |  Yes  No |
| 1. Remove triggers for challenging behaviors:  - When Jie sit down, mom just focus on guide him to start working. - Mom did not provide any attention or comments to Jie’s challenging behavior, e.g., when Jie asks for a hug or say, “ice cream” during the homework, mom does not say “no hug, no ice cream or you have eaten it”, when Jie cries, mom does not say “no crying”. |  Yes  No   Yes  No   Yes  No |
| 1. Provide a warning to inform the child of follow-up activities:  - Before the homework, Mom shows and reads Jie today’s homework tasks to Jie. |  Yes  No   Yes  No |
| Teach strategy |  |
| 1. Teach social skills – following rules:  - During the last 1 minute for transition, Mom shows the rule of homework, and the first rule is sat down at the study desk and chair set, and then prompt Jie to come to the worktable and sit down. - When Jie sit down, Mom reviews the rest rule of homework: Be quiet, look at the textbook, and follow mom’s instruction. - During the homework, prompt him be quiet when he is yelling, prompt him to look at his homework when he looks other way, prompt him to sit on the desk to work when he is leaving |  Yes  No   Yes  No   Yes  No   Yes  No |
| 1. After each task of the homework routine, prompt Jie through determining what was just done and what comes next.  - Use comments such as: - Ok, what’s first? - What did we just do? - Where do we put the picture after we’re done____? - What’s next? - Now what? - Provide support as needed for Jie to use visual schedule and answer the questions related to the homework routine. |  Yes  No   Yes  No   Yes  No |
| Reinforce strategy: |  |
| 1. When Jie follows the homework rule, provide descriptive praise to Jie, for example, “you did a great job on doing the work quietly”.  - Focus on Jie’s appropriate behavior during the homework time, and always praise the appropriate behavior with stick, thumbs up, verbal praise or hand heart. |  Yes  No   Yes  No |
| 1. When Jie finished the homework, mom hug him for a while and say, “you did a great job on finishing the homework, it’s time for mom to hug you now!” |  Yes  No |
| 1. Do not say “good job, Jie” when he engaged in some challenging behavior, e.g., crying, leaving.   If you cannot ignore challenging behavior, use neutral prompt back to schedule. Reinforce re-engagement quickly.   - Always repeat the rule and the schedule to Jie when he has challenging behavior if you cannot ignore his challenging behavior, do not say “No” or provide any other negative comments to him to trigger him. |  Yes  No   Yes  No   Yes  No |

**Dyad 2** Yiyi and Minyue

| **PTR-F Fidelity of Strategy Implementation Form** | |
| --- | --- |
| Child: Yiyi  Date: | Routine: Play with mom on the table  Person implementing: Mom |
| Strategy steps | Were the steps implemented as intended? |
| Prevent strategy: |  |
| 1. Timer for transition:  - 5 minutes before it is time for Yiyi to begin play with mom, Mom will make sure to have Yiyi’s attention and tell him “5 more minutes until play with mom. I’m going to set the timer.” The timer will be kept out of Yiyi’s reach. |  Yes  No   Yes  No |
| - 3 minutes before it is time for Yiyi to begin play with mom, Mom will make sure to have Yiyi’s attention and tell him “3 more minutes until play with mom. I’m going to set the timer.” The timer will be kept out of Yiyi’s reach. |  Yes  No |
| - 1 minutes before it is time for Yiyi to begin play with mom, Mom will make sure to have Yiyi’s attention and tell him “1 more minute until play with mom. I’m going to set the timer.” The timer will be kept out of Yiyi’s reach. - During this time, also show the rule of play with mom, and the first rule is sat down at the study desk and chair set, and then prompt Yiyi to come to the worktable and sit down. |  Yes  No   Yes  No |
| - When the timers up and the timer is beeping, Mom will take the timer to Yiyi and ask, “What time is it? It’s time to get ready for play with mom.” |  Yes  No |
| 1. Modify what is explicitly asked of the child:  - Mom could ask Yiyi to help (turn off the timer, bring something to mom, using some surprise voice or other task) instead of just saying “Yiyi, come here”. - Here mom remember, try your best to guide Yiyi come to the work area instead of just grab him (push him to the work area). - Mom could say “put it on the floor” instead of saying “put it away” - Mom could provide clear instruction, for example, put all red together, instead of saying “Categorize them”. |  Yes  No   Yes  No   Yes  No   Yes  No   Yes  No |
| 1. Provide choice  - Do Yiyi’s work schedule with Yiyi together. Mom can put a challenging task card, or a task mom want Yiyi must do in the second position on the My Schedule board and let Yiyi choose the first and third activities and sticker the activities cards to the My Schedule board (Note: the first task should not be challenging or nonpreferred for Yiyi). - Use comments such as: - Yiyi let's play together. How many activities do you want to do today? Show him that the options mom prepared, we have these activities to choose from. - What do you want to do first? You can choose one here, and then we will put it on your schedule together. When Yiyi chooses it himself, mom immediately praise him for his choice, “Yiyi made a good choice, good job!” - Okay, then we will do it (the one Yiyi chose) and then we will do (the second activity). - What else does Yiyi want to do after (the second activity)? You can choose one more here, and then we will sticker it on your schedule together. When Yiyi chooses it himself, mom immediately praise him for his choice, “good job on making choice!” |  Yes  No   Yes  No   Yes  No |
| 1. Enhancing predictability with schedules:   After finishing the work schedule with Yiyi, put it in a prominent place where Yiyi can see it at any time.   - At the beginning of the work routine, remind Yiyi to check the schedule, "Yiyi, what should you do first now?" - If no response from Yiyi, Mom will support Yiyi, e.g., point the card to Yiyi and prompt Yiyi to repeat the first activity. |  Yes  No |
| 1. Provide a warning to inform the child of follow-up activities:   -During a game/activity, mom could always let him know when the game/activity will be done. For example, if he is playing shape puzzle, mom could say 5 more puzzles, then the first activity is all done then we could play (second activity), 3 more puzzles…, 2 more… 1 more…etc. |  Yes  No |
| Teach strategy |  |
| 1. Teach social skills – following rules:  - During the last 1 minute for transition, Yiyi mom shows the rule of play, and the first rule is sat down at the study desk and chair set, and then prompt Yiyi to come to the worktable and sit down. - When Yiyi sit down, Mom reviews the rest rule of homework: Look (attention), and Listen (mom’s instruction). - Every time Yiyi did not follow the rule, Mom will review the rule with Yiyi first to reminder him to follow the rule, and once he did it, provide immediately verbal praise to him using the sentence “Yiyi did a great job on looking, listening or sitting on the chair!”. |  Yes  No   Yes  No   Yes  No   Yes  No |
| 1. FCT- asking for break: At the beginning of the work session, tell Yiyi that anytime you need a break, you need to tell me.  - When Yiyi wants to leave the seat during work time, Mom shows Yiyi’s schedule and work rules first, and remind him “It’s time for work, and we need to sit on the chair to do it.” - If Yiyi still wants to leave, mom show Yiyi a “break” card to him and prompt him to say “break”, and explain to him, if you feel tired it’s Ok to take a break. But you need let mom know you need a break before you leave the seat to take a break. When Yiyi uses the “break” to ask for leaving the seat, Mom immediately reinforces it (e.g., thumps up, verbal praise), and then said, “you did a great job on asking for a break, now you can take a short break here.” - Set the timer for 1 minute and say, “you worked very hard, and you can take 1 minute break now” and show Yiyi the timer. Do not have Yiyi take a break away from the work area, mom can remove the work stimuli altogether during the break or ask Yiyi to stretch on the seat. Also, do not provide other activities during break. - 30 seconds before it is time for Yiyi to back his work routine, Mom will make sure to have Yiyi’s attention and tell him “30 more seconds for break, and then come back.” Briefly review the work visual schedule again with Yiyi and show Yiyi the timer. - When the 1 minute is over and the timer is beeping, Mom will take the timer to Yiyi and ask, “What time is it? It’s time to get ready for work.” Mom shows Yiyi’s schedule and work rules again, and remind him “It’s time for work, and we need to sit on the chair to do it.” |  Yes  No   Yes  No   Yes  No   Yes  No   Yes  No   Yes  No |
| Reinforce strategy: |  |
| 1. Focus on Yiyi’s appropriate behavior during the homework time, and always praise the appropriate behavior with verbal praise, M&M chocolate.  - Every time Yiyi does correct, Mom provides verbal praise. For example, if mom is teaching the rule of sit on the chair, Yiyi sits on the chair, Mom provides immediately verbal praise for Yiyi using the sentence “Yiyi did a great job on siting on the chair for working!”. |  Yes  No |

**Dyad 3** Wei and Xiaoxiao

| **PTR-F Fidelity of Strategy Implementation Form** | |
| --- | --- |
| Child: Wei  Date: | Routine: Runing  Person implementing: Mom |
| Strategy steps | Were the steps implemented as intended? |
| Prevent strategy: |  |
| 1. Timer for transition:  - 3 minutes before it is time for Wei to begin his running schedule, Mom will make sure to have Wei’s attention and tell him “3 more minute for (his first schedule), and then running time.” The timer will be kept out of Wei’s reach. |  Yes  No   Yes  No |
| - 2 minutes before it is time for Wei to begin his running schedule, Mom will make sure to have Wei’s attention and tell him “2 more minute for (his first schedule), and then running time.” The timer will be kept out of Wei’s reach. |  Yes  No |
| - 1 minutes before it is time for Wei to begin his running schedule, Mom will make sure to have Wei’s attention and tell him “1 more minute for (his first schedule), and then running time.” The timer will be kept out of Wei’s reach. During this time, also prompt Wei "cool down" and slowly stop the first activity and get ready to run. |  Yes  No |
| - When the timers up and the timer is beeping, Mom will take the timer to Wei and ask, “What time is it? It’s time to get ready to run.” |  Yes  No |
| 1. Provide choice:  - Before running, ask Wei how many minutes he wants to run today, “15 or 20?” and then set the timer for him. And then set the timer with Wei together. |  Yes  No |
| 1. Remove trigger:  - Mom can teach the running posture to Wei separately. Do not touch or interrupt him while he is running during the exercise routine. - Mom does not ask him whether he need to sing if he did not ask. - Mom does not always stand with Wei, give him a space and time to run by himself |  Yes  No |
| 1. Provide a warning to inform the child of follow-up activities:   - Mom always let him know “you already run 5 minutes, 10/5/3/1 more minute, then running is all done”. Count back in the 10 more second to Wei. |  Yes  No |
| Teach strategy |  |
| 1. Teach social skills – following rules:  - Before running, Mom shows and read the rule of exercise, keep safe and calm body and try your best, to Wei - When Wei is running, mom should praise him when he is safe and calm in 5, 10, 15 minutes (when mom warns Wei how many minutes for running) |  Yes  No   Yes  No   Yes  No |
| Reinforce strategy: |  |
| 1. When Wei follows the exercise rule, especially, keep safe and calm body, provide immediately descriptive praise to Wei, for example, “you did a great job on keeping safe and calm body”.  - Focus on Wei’s appropriate behavior during the exercise time, and always praise the appropriate behavior with thumbs up, verbal praise. |  Yes  No |
| 1. Mom did not provide too many comments to Wei’s challenging behavior, when Wei cries, mom does not say “no crying”.  - If Wei did not keep safe and calm body, mom did not say “Do not XXX”, mom could repeat the rule “Keep safe and calm body”.   If you cannot ignore challenging behavior, use neutral prompt back to schedule. Reinforce re-engagement quickly. |  Yes  No   Yes  No |

**Dyad 4** Lanlan and Ting

| **PTR-F Fidelity of Strategy Implementation Form** | |
| --- | --- |
| Child: Lanlan  Date: | Routine: Play with mom  Person implementing: Mom |
| Strategy steps | Were the steps implemented as intended? |
| Prevent strategy: |  |
| 1. Provide choice:  - Before starting an activity, provide Lanlan with two options with visual cards to choose from, as this can help prevent overwhelm. For example, Mom could say, “Would you like to play with the blocks or the puzzles?” - If she cannot select with verbal language, ask her to pick one card. |  Yes  No   Yes  No   Yes  No |
| 1. Reduce distractions and materials  - When play a game, do not play too long (more than 15 minutes) provide too much, make the task easier to Lanlan engaged. - For example, Matching game, do not match more than 10 cards each time, and every time provide one or two cards to Lanlan to select to match, do not provide all cards (more than 10 cards) and ask Lanlan to select. - Another example is alphabet game, do not provide 26 alphabets to her at one time, one by one to ask her and always only provide less than 5 options to ask, “please give me letter M”. |  Yes  No |
| 1. Using timer for break:  - Use the timer for Lanlan’s break if she asked during the session, and warning her how many minutes/second lefts: - Use sentence such as: - You have 1 minute to take a break and then you need to come back - 30 more seconds for break then we need to keep playing - Count down from 10 while the timer has 10 more second. |  Yes  No   Yes  No |
| 1. Provide a warning  - During a game/activity, mom could always let him know when the game/activity will be done. For example, if he is playing shape puzzle, mom could say 5 more puzzles, then the first activity is all done then we could play (second activity), 3 more puzzles…, 2 more… 1 more…etc. |  Yes  No |
| 1. Modify what is explicitly asked of the child  - When play a higher-level game, provide clearer request. For example, do not just say “match”, try say a specific characteristic of the card to support Lanlan match the correct card. |  Yes  No |
| 1. Change how instructions are delivered:  - Instead of saying “No”, say “try again” - Instead of physical support, try verbal and visual support first - Do not speak too fast, be too anxious (e.g., keep calling Lanlan, and push her hurry up) |  Yes  No |
| Teach strategy |  |
| 1. FCT- asking for break: At the beginning of the work session, tell Lanlan that anytime you need a break, you need to tell me.  - When Lanlan wants to leave the seat during work time, Mom shows Lanlan’s schedule and work rules first, and remind her “It’s time for work, and we need to sit on the chair to do it.” - If Lanlan still wants to leave, mom show Lanlan a “break” card to her and prompt her to say “break”, and explain to her, if you feel tired it’s Ok to take a break. But you need let mom know you need a break before you leave the seat to take a break. When Lanlan uses the “break” to ask for leaving the seat, Mom immediately reinforces it (e.g., verbal praise), and then said, “you did a great job on asking for a break, now you can take a short break here.” - Set the timer for 1 minute and say, “you worked very hard, and you can take 1 minute break now” and show Lanlan the timer. Do not have Lanlan take a break away from the work area, mom can remove the work stimuli altogether during the break or ask Lanlan to stretch on the seat. Also, do not provide other activities during break. - 30 seconds before it is time for Lanlan to back her play with mom routine, Mom will make sure to have Lanlan’s attention and tell him “30 more seconds for break, and then come back.” - When the 1 minute is over and the timer is beeping, Mom will take the timer to Lanlan and ask, “What time is it? It’s time to come back for play with mom.” |  Yes  No |
| Reinforce strategy: |  |
| 1. Focus on Lanlan’s appropriate behavior during the Play time, and always praise the appropriate behavior with verbal praise, for example, “you did a great job on looking/listening”. |  Yes  No |
| 1. Mom did not provide any complain comments to Lanlan’s behavior, “Lanlan, why you are not looking/listening, why you are running away”. |  Yes  No |

**Dyad 5** Ningfeng and Nana

| **PTR-F Fidelity of Strategy Implementation Form** | |
| --- | --- |
| Child: Ningfeng  Date: | Routine: work with mom  Person implementing: Mom |
| Strategy steps | Were the steps implemented as intended? |
| Prevent strategy: |  |
| 1. Provide choice  - Do Ningfeng’s work schedule with him together. Mom can put a challenging task card, or a task mom want Ningfeng must do in the second position on the My Schedule board and let Ningfeng choose the first and third activities and sticker the activities cards to the My Schedule board (Note: the first task should not be challenging or nonpreferred for Ningfeng). |  Yes  No |
| 1. Enhancing predictability with schedules:   After finishing the work schedule with Ningfeng, put it in a prominent place where Ningfeng can see it at any time.   - At the beginning of the work routine, remind Ningfeng to check the schedule, " Ningfeng, what should you do first now?" - If no response from Ningfeng, Mom will support Ningfeng, e.g., point the card to Ningfeng and prompt Ningfeng to repeat the first activity. |  Yes  No |
| 1. Remove triggers for challenging behaviors: Mom does not push Ningfeng, Mom does not force to grab Ningfeng’s hand to come over, Mom does not yell “No” to him, Mom does not grab the toy in his hand.  - If Ningfeng leaves Mom could count back from 10 and then wait him to come over by himself |  Yes  No |
| 1. Before the work, limit the number of materials on the table, keep the play table area is clear, not provide all materials on the table. In addition, remove the materials which is not related to this routine and easier distract. (e.g., toy car). |  Yes  No |
| 1. When the mother gives instructions, the tone and intonation can be slightly exaggerated to attract Ningfeng 's attention, and don't just say "Come on, Ningfeng, read this, write this". You can say "Let's do it together..." Do not just request him to do something, inviting him friendly to join your activity. (no angry face and voice here) |  Yes  No |
| 1. Provide a warning to inform the child of follow-up activities: During a game/activity, mom could always let him know when the game/activity will be done. For example, if he is working on writing numbers, mom could say write 3 /2 /1 more numbers, then this activity is all done. |  Yes  No |
| Teach strategy |  |
| 1. Teach social skills – following rules:  - Before working, Mom shows the rule of homework, and the first rule is sat down at the study desk and chair set, and then prompt Ningfeng to come to the worktable and sit down. - When Ningfeng sits down, Mom reviews the rest rule of work: Be quiet, look at the table, and follow mom’s instruction. - During the work, prompt him to look at the task when he looks other way, prompt him to sit on the desk to work when he is leaving |  Yes  No |
| 1. After each task of the work routine, prompt Ningfeng through determining what was just done and what comes next.  - Use comments such as: - Ok, what’s first? - What did we just do? - Where do we put the picture after we’re done____? - What’s next? - Now what? - Provide support as needed for Ningfeng to use visual schedule and answer the questions related to the work routine. |  Yes  No |
| Reinforce strategy: |  |
| 1. Focus on Ningfeng’s appropriate behavior during the homework time, and always praise the appropriate behavior with verbal praise.  - Do not say “good job” without any reason. For example, Ningfeng is leaving, do not say “good job, Ningfeng”. |  Yes  No |
| 1. When Ningfeng is leaving, mom does not follow him, just stay in the space, and keep prompt him to come back.  - Always repeat the rule and the schedule to Ningfeng when he has challenging behavior if you cannot ignore his challenging behavior, do not yell “No” or provide any other negative comments to him to trigger him, do not hold him (physical restrict). |  Yes  No |

**Dyad 6** Meisheng and Yuanyuan

| **PTR-F Fidelity of Strategy Implementation Form** | |
| --- | --- |
| Child: Meisheng  Date: | Routine: work with mom  Person implementing: Mom |
| Strategy steps | Were the steps implemented as intended? |
| Prevent strategy: |  |
| 1. Enhancing predictability with schedules:  - Before the play/work, Mom shows Meisheng today’s activity to Meisheng one by one (e.g., write numbers, letters and name, fishing game or jump on the yoga ball), and ask Meisheng to stick the task cards on his work schedule one by one. |  Yes  No |
| 1. Remove triggers for challenging behaviors: Mom did not yell Meisheng when he has challenging behavior, do not discuss what happed with him when he has calm down e.g., when Meisheng throws his book, mom do not yell “No throwing”, just wait a second, if he calms down, and ask him to pick the book up. After his tantrum behavior, mom just ask him “are you ready to work?” Do not yell him and discuss about him “why you angry?” Mom keeps quiet, and do not provide any attention or comments during his challenging behavior, give him a space and time. |  Yes  No |
| Teach strategy |  |
| 1. Teach social skills – following rules:  - Before start, Mom shows the rule to Meisheng, and the first rule is sat down at the study desk and chair set, and then prompt Meisheng to come to the worktable and sit down. - When Meisheng sits down, Mom reviews the rest rule of work: look at the table, follow mom’s instruction, and keep safe and calm body. - During the work, prompt him to look at the task when he looks other way, prompt him to sit on the desk to work when he is leaving, when he engaged in tantrum behavior, mom could prompt him calm down first. |  Yes  No |
| 1. After each task of the work routine, prompt Meisheng through determining what was just done and what comes next.  - Use comments such as: - Ok, what’s first? - What did we just do? - Where do we put the picture after we’re done____? - What’s next? - Now what? - Provide support as needed for Meisheng to use visual schedule and answer the questions related to the work routine. |  Yes  No |
| Reinforce strategy: |  |
| 1. When he engaged tantrum behavior, mom does not try to meet his requirement. For example, “tell me what you want?”, “Do you want goldfish or gummy?”, “Ok, we will not do XXX”.  - Always repeat the rule and the schedule to Meisheng, do not yell “No” or provide any other negative comments to him to trigger him, do not hold him (physical restrict). - Reinforce re-engagement quickly. |  Yes  No |
