# Supplementary material for: Remote delivery of culturally adapted prevent-teach-reinforce for families with Chinese American families of young autistic children
Source: Front Psychiatry. 2026 Apr 21;17:1783825. doi: 10.3389/fpsyt.2026.1783825 (PMC13140853; doi:10.3389/fpsyt.2026.1783825)
Supplement: Supplementary file 1 [file SupplementaryFile1.zip › Supplementary Table 2 Training Fidelity Checklist.docx]

# **Training Fidelity Checklist**

Date: __________________ Start time: ______________ End time: ________________

Participant code: __________

*Training protocol checklist*

| Phase | Steps | Were the steps implemented as intended? |
| --- | --- | --- |
| Instruction | Review: all the strategies the parents selected for their children |  Yes  No |
|  | Introduction: explain the strategies they selected one by one |  Yes  No |
|  | Teaching: present the steps of teaching each strategy |  Yes  No |
|  | Fidelity checklist: was provided |  Yes  No |
|  | Encouragement of questions: from parents |  Yes  No |
| Modeling | Demonstration of targeted skill |  Yes  No |
|  | Addressing potential difficulties |  Yes  No |
|  | Encouraging questions |  Yes  No |
| Rehearsal | Provided questions for parents practice |  Yes  No |
|  | Asked parents to retell the implementation checklist |  Yes  No |
|  | Reviewed one baseline video with some questions |  Yes  No |
| Feedback | Positive comments |  Yes  No |
|  | Corrected they made an error |  Yes  No |
|  | Answered for parent’s answer |  Yes  No |
|  | Reviewed the step they struggled with and asked if they have any other questions or want to practice more after the feedback |  Yes  No |
