# Supplementary material for: Remote delivery of culturally adapted prevent-teach-reinforce for families with Chinese American families of young autistic children
Source: Front Psychiatry. 2026 Apr 21;17:1783825. doi: 10.3389/fpsyt.2026.1783825 (PMC13140853; doi:10.3389/fpsyt.2026.1783825)
Supplement: Supplementary file 1 [file SupplementaryFile1.zip › Supplementary Table 3 Coaching Fidelity Checklist.docx]

# **Coaching Fidelity Checklist**

Date: _________________ Start time: ______________ End time: ________________

Participant code: __________

|  | Completed? | |
| --- | --- | --- |
| 1. Interventionist begins the session by greeting and briefly stating agenda and timeline. |  Yes |  No |
| 2. Observe caregiver/child interaction in the target routine without interruption |  Yes |  No |
| Performance feedback |  |  |
| 3. Have caregiver reflect on interaction before providing feedback: *How did you feel when you used the strategies?* |  Yes |  No |
| 4. Praise for the behavior the parents performed correctly: *You did a great job on…* |  Yes |  No |
| 5. Point the behavior the parents performed incorrectly: *But I notice that …* |  Yes |  No |
| 6. Provide a rationale for changing behavior performed incorrectly |  Yes |  No |
| 7. Provide the instructions for correct performance |  Yes |  No |
| 8. Demonstrate correct performance |  Yes |  No |
| 9. Provide an opportunity for the parents to practice correct performance |  Yes |  No |
| 10. Provide an opportunity for the parents to ask questions |  Yes |  No |
| 11. Coaching session ends with the interventionist summarizing the primary caregiver’s positive growth in knowledge and use of targeted skill, and emphasize next session’s target skill. |  Yes |  No |
| **Percentage of steps completed correctly during coaching session** |  |  |
