# Supplementary material for: Remote delivery of culturally adapted prevent-teach-reinforce for families with Chinese American families of young autistic children
Source: Front Psychiatry. 2026 Apr 21;17:1783825. doi: 10.3389/fpsyt.2026.1783825 (PMC13140853; doi:10.3389/fpsyt.2026.1783825)
Supplement: Supplementary file 1 [file SupplementaryFile1.zip › Supplementary Table 4 Mothers' satisfaction with the telepractice delivery modality .docx]

**Online Supplemental Table 7** Mothers' satisfaction with the telepractice delivery modality

|  | Jie and his family | Yiyi and his family | Wei and his family | Lanlan and her family | Ningfeng and his family | Meisheng and his family | Mean |
| --- | --- | --- | --- | --- | --- | --- | --- |
| Prior to interacting with the therapist and conducting sessions, you were required to set up several pieces of technology. I was satisfied with the process of accessing the Zoom meeting room and starting my audio and webcam? | 7 | 7 | 6 | 7 | 7 | 7 | 6.83 |
| In order for us to evaluate your performance, you were video recorded while conducting sessions. I was satisfied with the process of recording the sessions. | 7 | 7 | 6 | 7 | 7 | 7 | 6.83 |
| I was you satisfied with the audio and video quality of the remote interactions (via Zoom). | 7 | 5 | 7 | 7 | 7 | 7 | 6.67 |
| We conducted virtual meetings, using PowerPoint, to review the goals for the upcoming week and provide a status update. I was satisfied with the quality and organization of the PowerPoint presentation. | 7 | 7 | 7 | 6 | 7 | 7 | 6.83 |
| Virtual meetings were also conducted for teaching during role-plays. I was satisfied with the reliability (e.g., consistent internet connection) and the audio and video quality during the role-plays. | 7 | 6 | 7 | 7 | 7 | 7 | 6.83 |
| I think this process of remote instruction  would be acceptable for teaching other skills relevant to your child’s treatment? | 7 | 7 | 6 | 7 | 7 | 7 | 6.83 |
| Overall, I was satisfied with this virtual-training program. | 7 | 7 | 6 | 7 | 7 | 7 | 6.83 |
| Would you recommend remote instruction to other individuals who are not able to receive on-site training? | 7 | 7 | 7 | 7 | 7 | 7 | 7 |
| Total score | 56 | 53 | 52 | 55 | 56 | 56 | 55.17 |
